# The Lifetime of Experiences Questionnaire: Psychometric Properties and Relationships With Memory Function in an Iranian Elderly Sample

**DOI:** 10.3389/fpsyt.2022.889177

**Published:** 2022-05-02

**Authors:** Hossein Karsazi, Javad Hatami, Reza Rostami, Ali Moghadamzadeh

**Affiliations:** ^1^Department of Psychology, Faculty of Psychology and Educational Sciences, University of Tehran, Tehran, Iran; ^2^Department of Curriculum Development and Instruction Methods, Faculty of Psychology and Educational Sciences, University of Tehran, Tehran, Iran

**Keywords:** Lifetime of Experiences Questionnaire, older adults, cognitive decline, Persian version, memory

## Abstract

This study aimed to validate the Persian version of the Lifetime of Experiences Questionnaire (LEQ) and examine the relationship between life experience and memory function. To this end, two studies were conducted. Study one examined the factor structure, internal consistency, and convergent and discriminant validity of the Persian version of LEQ with 247 healthy elderly individuals (M age = 70.17, *SD* = 4.42; 55.9% women). The exploratory factor analysis yielded a five-factor solution, including Knowledge, Physical, Socialization, Artistic, and Leisure dimensions, which accounted for 42.42% of the explained variance. The internal consistency of the LEQ was in the acceptable range (α = 0.703). Also, the LEQ and its subscales (except the Artistic subscale) had a negative relationship with geriatric depression and a positive relationship with a healthy lifestyle, supporting the measure's convergent and discriminant validity. In the second study, we examined the relationship between LEQ (total and subscales score) and memory function using Pearson correlation and moderating analysis with 149 participants (M age = 70.37, *SD* = 4.29; 55.03% women). The results demonstrated that the LEQ total and subscales scores correlated positively with episodic memory. In the same vein, LEQ total score and Knowledge subscale showed a positive correlation with semantic memory. The results of moderation analysis showed that LEQ subscales have a different role in memory decline. The knowledge subscale as a content component moderates the effect of age on semantic memory (B = 0.005, *t* = 2.021, *p* = 0.045), while the mid-life non-specific activities, which are based on life stages, moderate the effect of age on episodic memory (B = 0.007, *t* = 3.348, *p* = 0.001). In conclusion, our results indicated that the Persian version of the LEQ is a valid and reliable instrument for measuring experiences and activities throughout life, which can be used in professional clinical and research settings in the aging context with Iranian elderly samples. Furthermore, our findings suggest that various life experiences can be considered cognitive reserves in old age.

## Introduction

It is acknowledged that worldwide, the population is aging rapidly, and the proportion of the elderly is growing more and more. The aging process has become one of the main challenges of our time and has led not only to economic and social problems but also to medical and psychological difficulties (1). While all organs are influenced by aging, cognitive impairment is one of the most impaired outcomes of aging in day-to-day activities and one of the most prevalent complaints of older adults (2).

Cognitive aging is characterized by declines in various cognitive abilities; however, trajectories of aging demonstrate substantial individual differences. Some people show a high degree of deterioration, whereas others preserve their cognitive performance during aging (3). According to recent estimates, on average, only about 50% of interindividual variability in cognitive decline can be explained by age-related pathologies (4, 5), suggesting that other factors may also influence cognitive trajectories in individuals. Findings in this field have led to the hypothesis that some individuals may develop a reserve that permits them to tolerate age-related changes. To explain this finding, researchers have used various constructs, including brain reserve (6, 7), cognitive reserve (8), brain maintenance (9), and adaptive brain (10).

The origins of the development and continuity of this reserve are still unclear. However, this reserve seems to be influenced by the experiences in different periods of life (8). Different enriching experiences, such as education, occupational attainment, leisure, and social activities are associated with better cognitive function in the face of aging and brain pathologies (11, 12). Furthermore, due to the lack of effective treatments for Alzheimer's disease (AD), new therapeutical approaches focus on preventing and delaying the onset of AD symptoms (13, 14). Life experiences such as physical, social, and cognitive activity are essential for preventing AD (15, 16). Also, modification of lifestyle routines can reduce the incidence of dementia by as much as 35% (13, 14).

Various assessment instruments have been developed to measure engagement in cognitively stimulating activities and life experiences. However, most of these tools are cross-sectional scales (17–20). Engagement in activities may differ across different stages of life, which is an essential subject in the context of aging. Older adults who have been actively involved in socially and cognitively stimulating activities throughout their lives withdraw gradually from these activities due to their physical problems. Ignoring this issue can lead to bias in assessing stimulating activities and affect their relationship with cognitive performance in late adulthood (21). Therefore, cross-sectional scales may not fully represent lifelong activities.

In this regard, several assessment tools have been developed to measure activities across different stages of life (22–24). Among these measures, the Lifetime of Experiences Questionnaire (LEQ) (24) is one of the earliest and most comprehensive instruments of lifetime cognitive activity that assesses a broad range of cognitively stimulating activities during three life stages, including young adulthood, mid-life, and late-life (25). LEQ is only administrable to individuals over 65 years of age. The LEQ was initially developed by Valenzuela and Sachdev (24) in Australia and was validated in several countries, including the United States (26), Germany (27), and India (28) and harmonize across Spain, United Kingdom, France, and Germany (29).

Valenzuela and Sachdev (24) reported that the LEQ is a reliable and valid instrument for assessing complex lifespan mental activities, which are protective against cognitive decline. In the same vein, Gonzales (26) demonstrated that the LEQ had good temporal stability and acceptable test-retest reliability, but the concurrent validity was not satisfactory. In another study, Paplikar et al. (28) showed that the LEQ had high inter-rater and test-retest reliability and found acceptable internal consistency for total and sub-scores. Reports on the measure's reliability show Cronbach's alpha ranging from 0.43 to 0.84 for subscales, and the total is 0.66. The overall level of test-retest reliability was high (*r* = 0.98). Concurrent validity of the LEQ was confirmed through the zero-order intraclass correlation between total LEQ and Cognitive Activities Scale (CAS) (*r* = 0.41) and change in performance (*r* = 0.37) (24).

The LEQ has also been used in several studies to estimate the relationship between life experience and cognitive function. LEQ total score was positively associated with better executive functions (30) and better functional status during the onset in dementia (31). The mid-life subscale of LEQ moderated the relationship between brain health and late-life cognitive ability, with the cognitive ability of people with higher mid-life activities being less dependent on their brain structure (32). Further, Opdebeeck et al. (33) showed that a higher LEQ score was associated with better performance in delayed recall, immediate recall, and verbal fluency. Higher LEQ scores were also related to a higher quality of life, self-esteem and self-efficacy (34), and lower depressive symptoms (35) in later life.

Furthermore, evidence from brain research suggests that higher LEQ scores are associated with a reduced rate of hippocampal atrophy (36). For instance, Suo et al. (37) used the LEQ and demonstrated that cognitive lifestyle is generally related to hippocampal volume in late life. In the same vein, Kinney et al. (38) examined the association between social engagement and leisure activities on the LEQ and cortical thickness (CT). Their findings revealed that higher social engagement and leisure activity were associated with greater CT in the superior anterior temporal region, including the amygdala. Another study reported that a higher late-life LEQ score was associated with less atrophy in the left superior and inferior anterior temporal regions and the right middle temporal gyrus (39). These findings suggest that life experiences may provide a kind of resilience against cerebral atrophy.

Despite the widespread use of LEQ in cognitive studies, this tool suffers from some validation challenges. Although it enjoys good convergent validity, reliability, responsiveness, and fair content validity, information regarding the factor structure of the LEQ is too scarce, and no conclusions can be drawn in this regard (25). This uncertainty in the factor structure of the LEQ may be due to methodological issues. To our knowledge, only two studies have examined the factor structure of LEQ (24, 26). Both studies used explanatory factor analysis and an eigenvalue equal to or > 1 (Kaiser criterion) to retain the factors. This criterion leads to the keeping of many factors that may conflict with the principle of parsimony (40). As a result, 20 factors were retained in Valenzuela and Sachdev (24) study and 9 in Gonzales (26) study. Therefore these number of factors make the interpretability of the factor structure challenging.

Several studies have shown that the Kaiser criterion is inaccurate and overestimates the number of factors (41–44). A proper alternative to the Kaiser criterion is parallel analysis (PA). PA attempts to overcome a primary limitation of the Kaiser criterion (45). Numerous studies on real and simulated data show that parallel analysis is the most accurate approach to identify the number of factors in factor analysis (41, 46). Given the evidence, researchers should consider PA as a primary method for factor retention decisions in EFA of LEQ and should also rely less on the Kaiser criterion.

While LEQ is a widely used research measure in Western countries, an appropriate measure for assessing lifetime experiences is unavailable in Iran. Thus, there is a clear need to validate established measures of life experiences for researchers to investigate lifetime experiences in Iranian older adults. Furthermore, there is a need to understand the role of protective life experience, specific to the Iranian context, which contributes to better memory performance in late life. Also, due to the lack of content categorization of LEQ items, previous studies have focused on the relationship of stage-based subscales of this tool with cognition, and the effect of the different categories of activities on cognitive function has not been studied before. The categorization of these activities also allows comparing the distinct role of the type of activities (content-based subscales) vs. life stages (stage-based subscales) in the cognitive function of old age.

However, since the LEQ has not been validated in Iran, we first examine its psychometric properties. Then, we will proceed with the correlation and moderation analysis, which will allow us to determine the influence of life experiences on the memory function. Therefore, this study includes two studies: In the first study, we examined the factor structure, reliability, convergent, and discriminant validity of the Persian version of the LEQ. We used PA to identify the optimal number of factors in this questionnaire. To examine the reliability of the Persian LEQ scores, reliability indices, including Chronbach's Alpha (α) and mean inter-item correlation (MIC) values, will be calculated. In order to investigate the convergent and discriminant validity of the LEQ scores, the correlations between these scales and scores of the Yonsei Lifestyle Profile (YLP) and the Geriatric Depression Scale Short-Form (GDS-SF) will be calculated. Specifically, it is hypothesized that lifetime experiences would positively relate to a healthy lifestyle and negatively to depression. With respect to the second study, we hypothesized that lifetime experiences (content-based and stage-based subscales) correlate with memory performance in the healthy elderly and moderate the relationship between age and memory.

## Study 1

This study aimed to examine the factor structure, internal consistency, and convergent and discriminant validity of the Persian version of the LEQ.

## Materials and Methods

### Procedure

LEQ was translated to Persian by two fluent English and Persian translators. Afterward, Persian translations were translated back from Persian to English by a third, independent translator. Then we implemented comments by three specialists in psychology for reviewing and revising the questionnaire. Furthermore, we recruited 10 older adults and requested them to complete the LEQ and declare any problems, questions, or misinterpretations about the clearness and structure of the items. We modified the challenging statements based on their feedback to make them clear and more transparent. Regarding the sample size calculation in the exploratory factor analysis, recommendations are based on the number of items. The researchers generally suggested having at least a 5:1 ratio of participants to an item (47); however, having a 10:1 ratio is more acceptable (48, 49). The sample size in this research was estimated based on the 8:1 ratio, which falls within acceptable limits (50, 51).

Participants were recruited *via* advertisements on social networks that provided information about the study. The study took place in the community of Tehran, Iran, from January 2021 to June 2021. Data was gathered either at participants' homes or any suitable place agreed upon by the participants and the researcher. The inclusion criteria were: (a) at least 65 years of age and older, (b) being able to speak and read in Persian, and (c) no history of brain disorders (e.g., stroke, traumatic brain disorder), neurological illness, as well as visual and auditory impairments. The written informed consent was received from the participants meeting the inclusion criteria before answering the questionnaires.

### Participants

This study was conducted with a sample of 247 healthy elderly individuals (aged 65 to 84; M-age = 70.17 years; *SD* = 4.42; 55.9% women) who were recruited through the purposeful and convenience sampling method. The 69.9% of the participants were married, 2% were single, 6.5% divorced, and 21.9% had lost their spouse. The educational level of the participants was as follows: 10.5% with a Ph.D., 25.9% with a Master's degree, 31.6% with a Bachelor's degree, 12.1% with an advanced diploma degree, 15.0% with a High School Diploma, and 4.9% did not attend graduate high school.

### Instruments

#### The Lifetime of Experiences Questionnaire

The LEQ was first developed by Valenzuela and Sachdev (24) to comprehensively measure the educational, occupational, cognitive, and leisure activities at different stages through life. The questionnaire is divided into three stages, namely, young adulthood (13–30 years), mid-life (31–65 years), and late-life (65 years onwards). Within each phase, activities are subdivided into specific and non-specific. A set of non-specific questions is the same for all life stages and assesses the frequency of participants' engagement in seven activities*:* (1) visiting family/friends, (2) playing a musical instrument, (3) artistic pastimes, (4) physical activity (mild, moderate, vigorous), (5) reading, (6) practice speaking, reading, writing or learning a second language, and (7) travel. On the original scale, the travel item included travel to foreign countries. In the translated version, we changed this item to travel to different states of Iran and the other regions/countries because most Iranians do not frequently travel outside the country due to their poor economic status and unstable sources of income. The non-specific items are rated on a 6-point Likert scale ranging from 0 = (*Never*) to 5 = (*Daily*). Specific activities are those that experienced at a particular life phase. The specific score of young adulthood assesses the level of education achieved before the age of 30. The mid-life score contains scores for occupational complexity and supervisory role between the ages of 30 and 65. The late-life-specific score reflected the frequency of engagement in social and intellectual activities such as membership in social clubs or groups, travel or participation in volunteer activities, and methods of seeking information about the world over the age of 65. Some specific items measure the type of activities, and some measure the frequencies. The greater variety and frequency of activities indicate high scores on the scale.

#### The Yonsei Lifestyle Profile

Park and Park (52) developed the YLP to measure three different lifestyle factors in the elderly: (1) physical activity, (2) participation in activities, and (3) nutrition. Respondents are asked about the frequency of their participation in certain activities or food consumption for a week and the number of times they spent certain activities during the day. In addition, satisfaction with their participation in activities and their consumption of nutrition were assessed. Except for the items about satisfaction, all other questions can be measured on a 5-point Likert scale. The satisfaction questions (e.g., “*Do you do as much as you want?”*) are measured on a 3-point Likert scale (1: always less/more than I want; 2: Sometimes less/more than I want; 3: about right for me), with higher total score showing more healthy and balanced the lifestyle. To obtain an indicator of an active lifestyle, we considered the frequency and satisfaction of each activity. For nutrition, only nutrition satisfaction was used in the analysis because the frequency of food consumption could not be an accurate indicator of a healthy lifestyle. The YLP enjoys high internal reliability, with a Cronbach's alpha of 0.83. The intraclass correlation coefficient was 0.97 for the total score of the YLP regarding test-retest reliability (53).

#### The Geriatric Depression Scale-Short Form

The GDS-SF is one of the most widely used instruments for measuring depression among the elderly. Its advantages over other depression scales are the ease and time-effectiveness of the administration. GDS-SF was developed by Sheikh and Yesavage (54) and includes 15 items that are rated by answering “Yes” or “No” about how one felt over the past week. Scores range from zero to 15. Scores of 0–4 reflect normal depression; 5–8 indicate mild depression; 9–11 indicate moderate depression; and 12–15 indicate severe depression. GDS-SF was validated by comparing the Long and Short Forms of the GDS for self-rating of symptoms of depression, and both were successful in differentiating depressed from non-depressed adults with a high correlation (*r* = 0.84, *p* < 0.001) (54). The Persian version of GDS-SF revealed good test-retest reliability (*r* = 0.58), split-half reliability (*r* = 0.89), and excellent internal consistency (α = 0.90) (55).

### Statistical Analyses

The EFA was utilized with principal component analysis (PCA) and varimax rotation in the first study. The suitability of the data for EFA was assessed by calculating the Kaiser-Meyer-Olkin Test for sample adequacy. Values close to 1 indicate high sample adequacy, and the acceptable range is 0.6 and above (56). Moreover, by assessing the inter-correlation of the items, Bartlett's Test of Sphericity evaluates the data's potential for factor extraction. The test should be significant at the *p* < 0.05 level (57). We used Comrey and Lee (58) cut-off point for factor loading, where values > 0.32 are the minimum acceptable threshold. The criterion for retaining the optimal number of components was per Parallel Analysis. Parallel analysis (43) is based on comparing eigenvalues of the real data to correspondent eigenvalues of the random data. In this process, the number of factors at the point where the eigenvalue in the real data is greater than that of the random data is considered significant. Cronbach's alpha coefficient was also used to examine the internal consistency of the components. Cronbach's alpha reliability coefficient ranges between 0 and 1, and the closer it is to 1.0, the greater the internal consistency of the items in the scale. George and Mallery (59) provide the following rules of thumb for Cronbach's alpha coefficient: “> 0.9 = Excellent; > 0.8 = Good; > 0.7 = Acceptable; > 0.6 = Questionable; > 0.5 = Poor; and 0.5 > = Unacceptable” (p. 231). In contrast to α, mean inter-item correlation (MIC) values are not dependent on the number of items in a scale and should be in the range of 0.15 to 0.50 to be considered adequate (60). The convergent and discriminant validity of the LEQ was assessed by estimating its bivariate correlations with the GDS-SF and YLP subscales scores. Correlation coefficients were interpreted as ≤0.30 = small; 0.30–0.50 = medium; and ≥0.50 = strong effect sizes (61). Statistical analyses were performed using SPSS software ver. 23.0.

## Results

### Exploratory Factor Analysis (Factor Structure)

**Figure 1:**
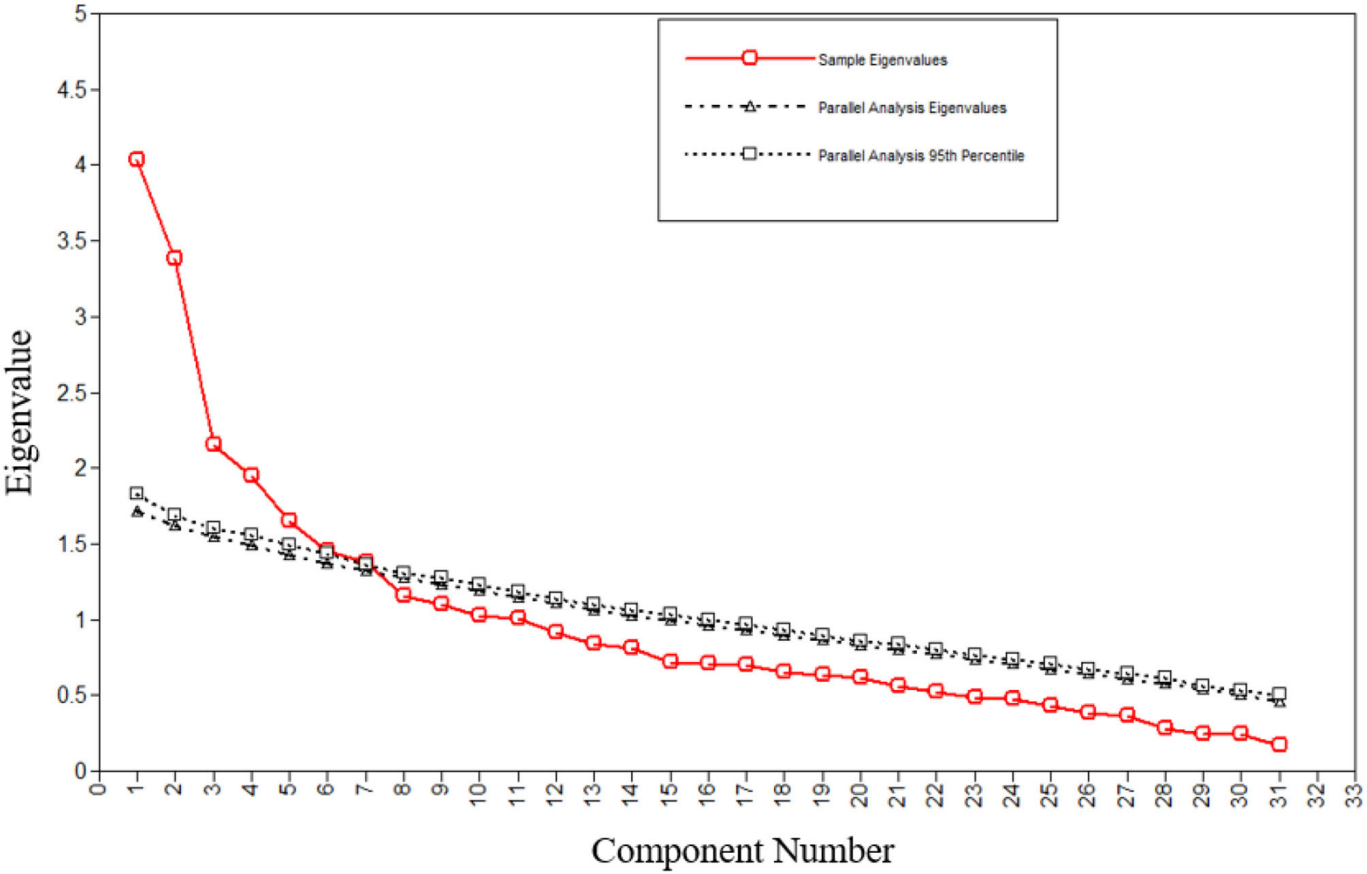
Parallel analysis of the LEQ items (*n* = 247).

Before performing the EFA, the suitability of the data for factor analysis was assessed. The Kaiser– Meyer–Olkin measure verified the sampling adequacy for the analysis (KMO = 0.677). Also, Bartlett's test of sphericity indicated that correlations between items were factorability for an EFA (χ2 = 2,059.38, *p* < 0.001). An initial analysis showed that 11 factors had eigenvalues greater than one and explained 65.33% of the variance. However, the parallel analysis suggested a 5-factor solution (Figure 1). The analysis was repeated with a forced 5-factor solution. The 5-factor solution accounted for 42.42% of the variance. Table 1 shows the factor loadings of the 5-factor solution after rotation.

**Table 1:** LEQ factor loadings, Cronbach's alpha, MIC, and correlation matrix for the 5-factor solution (*n* = 247).

**Item**	**Components**				
	**Knowledge**	**Physical**	**Socialization**	**Artistic**	**Leisure**
ML_ Reading	**0.686**				
YA_ Reading	**0.675**				
LL_ Reading	**0.606**				
ML_Language	**0.573**				
YA_ Language	**0.562**				
LL_ Language	**0.505**				
LL_Kinds of reading materials	**0.474**				
YA_Education score	**0.448**				
LL_ Information sources					
ML_ Physical activity		**0.813**			
YA_ Physical activity		**0.799**			
LL_ Physical activity		**0.735**			
LL_ Work		**0.339**			
ML_ Occupation rank					
ML_ Visiting			**0.786**		
LL_ Visiting			**0.742**		
YA_ Visiting			**0.677**		
LL_ Volunteer			**0.664**		0.387
LL_ Social club		0.341	**0.412**		
ML_ Supervisory role					
ML_ Artistic pastimes				**0.633**	0.349
YA_ Artistic pastimes				**0.576**	
LL_ Artistic pastimes				**0.514**	
ML_ Instrument				**0.501**	
YA_ Instrument	0.410			**0.495**	
LL_ Instrument				**0.443**	
ML_ Travel					**0.636**
YA_ Travel					**0.552**
LL_ Entertainment					**0.452**
LL_ Travel		0.417			**0.443**
LL_ Activity of daily living			0.403		**0.425**
Eigenvalues	3.283	2.873	2.676	2.257	2.063
% of Explained variance	10.591	9.269	8.631	7.280	6.654
Cronbach's alpha (Total = 0.703)	0.721	0.705	0.724	0.568	0.530
MIC	0.245	0.374	0.344	0.180	0.184
Knowledge	1				
Physical	0.108	1			
Socialization	−0.018	0.182^**^	1		
Artistic	0.192^**^	−0.018	−0.119	1	
Leisure	0.249^**^	0.277^**^	0.269^**^	−0.051	1

** *p < 0.01*.

*YA, young adulthood; ML, mid-life; LL, late-life; MIC, mean interitem correlation. The bold values indicate the corresponding items belonging to each component*.

The factor structure has considerable clarity. Most items have values above 0.32. The exception was LL_ Information Sources, ML_ Occupation Rank, and ML_ Supervisory Role, which had factor loading below the threshold. We decided to assign the cross-loading items to a higher factor loading component. We interpreted the five extracted components based on the research background and resources of activities. These components were named Knowledge, Physical, Socialization, Artistic, and Leisure. The Knowledge component includes eight items and explained 10.59% of the variance. It comprises reading materials and using a second language in all three stages of life, kinds of reading materials in late life, and young adulthood education. The Physical component comprises physical activity in all three stages and late-life work. This component contains four items and explained 9.27% of the variance. Visiting friends and family in all three stages, volunteer activities, and participating in social clubs in late-life formed the component of Socialization. The number of items for this component is five, and the variance explained is 8.63%. Artistic pastimes and playing music in all three stages were covered in the Artistic component. These six items accounted for 7.28% of the variance. Travel in all three stages, entertainment, and activity of daily living in late-life also formed the Leisure component. These five items also explained 6.65% of the variance of LEQ.

### Internal Consistency

Cronbach's alpha coefficients and mean inter-item correlations (MIC) were used to determine the internal consistency. As shown in Table 1, when using α as the measure of internal consistency, the values ranged from unacceptable (Leisure, α = 0.530) to acceptable (Socialization, α = 0.724). However, when relying on MIC values as the index of internal consistency, factors of Knowledge (0.245), Physical (0.374), Socialization (0.344), Artistic (0.184), and Leisure (0.184) had adequate internal consistency, but the MIC of the total score was not in the acceptable range (0.071), since MIC is susceptible to a high number of items. The correlation matrix between the components is also shown in Table 1. Inter-factor correlations ranged from −0.119 to 0.269.

### Convergent and Discriminant Validity

The results indicate that the LEQ yielded the expected positive correlations with similar constructs (convergent validity) and negative or insignificant correlations with conceptually unerelated constructs (discriminant validity). Regarding convergent validity, as shown in Table 2, scores on the LEQ had a positive correlation with a healthy lifestyle measured by YLP. LEQ Total score was highly associated with the total score of YLP (*r* = 0.873, *p* < 0.01). Physical and Leisure subscale has a high correlation with YLP (*r* = 0.731, *p* < 0.01; and *r* = 0.721, *p* < 0.01, respectively). Socialization and Knowledge subscales had moderate (*r* = 0.493, *p* < 0.01) and weak correlation (*r* = 0.185, *p* < 0.01) with YLP. The Artistic subscale did not show a significant relationship with the YLP (*r* = −0.044, *p* = ns). Among the life stages, the specific late-life activities were more related to the YLP (*r* = 0.741, *p* < 0.01) (Table 2).

**Table 2:** Bivariate correlations between LEQ scores, GDS-SF, and YLP (*n* = 247).

		**Subscales**	**GDS-SF**	**YLP**			
				**Physical activity**	**Activity participation**	**Nutrition satisfaction**	**Total YLP score**
LEQ	5-factor structure	Knowledge	−0.271^**^	0.091	0.209^**^	0.170^**^	0.185^**^
		Physical	−0.315^**^	0.641^**^	0.604^**^	0.360^**^	0.731^**^
		Socialization	−0.220^**^	0.393^**^	0.446^**^	0.257^**^	0.493^**^
		Artistic	−0.071	−0.036	−0.051	0.024	−0.044
		Leisure	−0.415^**^	0.509^**^	0.718^**^	0.409^**^	0.721^**^
		LEQ total score	−0.491^**^	0.678^**^	0.802^**^	0.486^**^	0.873^**^
	Original structure	YA specific	−0.169^**^	0.062	0.193^**^	0.112	0.151^*^
		YA non-specific	−0.404^**^	0.402^**^	0.454^**^	0.322^**^	0.511^**^
		ML specific	−0.158^*^	0.006	0.047	0.056	0.036
		ML non-specific	−0.355^**^	0.364^**^	0.443^**^	0.297^**^	0.480^**^
		LL specific	−0.443^**^	0.533^**^	0.717^**^	0.454^**^	0.741^**^
		LL non-specific	−0.200^**^	0.331^**^	0.344^**^	0.189^**^	0.394^**^

** *p < 0.01*;

* *p < 0.05*.

*LEQ, The Lifetime of Experiences Questionnaire; GDS-SF, The Geriatric Depression Scale-Short Form; YLP, The Yonsei Lifestyle Profile*.

Regarding discriminant validity, the LEQ total score was negatively correlated with The Geriatric Depression Scale (*r* = −0.491, *p* < 0.01). Except for the Artistic activities (*r* = −0.071, *p* = ns), the other four activities were significantly associated with geriatric depression (*r's* from −0.220 to −0.415). In terms of stage of activities, late-life-specific activities had the highest correlation with depression (*r* = −0.443, *p* < 0.01).

## Study 2

The second study examined the relationship between life experiences and memory function. We also tested the moderating role of life experiences in the relationship between age and memory.

## Materials and Methods

### Procedure

We conducted a Power analysis in G*Power (62) to determine the minimum required sample size for moderation analysis. A power analysis was done using an alpha level of 0.05, a power of 0.80, an effect size of 0.15, and three predictors (independent variable, moderator, and interaction). The results of this analysis suggested a minimum sample size of 55 participants. Thus, the sample size of the current study (*n* = 147) was deemed appropriate for moderation analysis. All participants provided written informed consent for their participation in this study. They first responded to the LEQ and then completed computerized memory tests. Assessments were done in various places but with the same protocol from July 2021 to October 2021 in Tehran, Iran. We used the same inclusion criteria used previously in the first study.

### Participants

The second study included 149 healthy elderly participants (Mage = 70.37 years; *SD* = 4.29; 55.03% women). Of these, 72.5% were married, 2.7% were single, 4.7% divorced, and 20.1% were widowed. The educational level of the participants was as follows: 10.1% with a Ph.D., 21.5% with a Master's degree, 34.2% with a Bachelor's degree, 11.4% with an advanced diploma degree, 18.1% with a High School Diploma, and 4.7% did not attend graduate high school.

### Instruments

In the second study, besides the Persian version of the LEQ, memory performance was measured. We used the computerized memory tests from the Sepidar cognitive Test battery (63–65). The battery was initially developed by Nilsson et al. (66) and was translated into Persian by Hatami et al. (63). The following tests were used in this study to measure episodic and semantic aspects of memory:

### Episodic Memory

#### Recalling of Imperative Sentences

This test consists of memorizing and recalling the two lists of 16 imperative sentences with/without action. For the list of action sentences (e.g., “*give me the pencil”*), participants were asked to memorize the sentences by performing the actions. In comparison, the second list was only encoded by the participant auditorily. The number of sentences recalled from each list was used as the measure in data analysis.

#### Cued Recalling of Imperative Sentence Nouns

The participants were provided with eight noun categories (toy, tools, stationery, kitchen utensils, clothing, edibles, home furniture, sewing tools). Each category consisted of four nouns included in the imperative sentences presented in the previous section. We asked participants to recall these nouns. The number of nouns recalled correctly was used in data analyses.

#### Recognition of Faces and Names

At the beginning of the assessment, participants were presented with 16 pictures of children's faces with full names and were asked to remember them for later recall. Approximately 30 mins after the start of the test, the participants were then presented with 24 faces (12 target and 12 distractor faces). They were asked to recognize the target images from the distractor ones. In another part of the test, the same 16 images of children were shown to the participants once again (target faces). For each picture, four options were provided for participants, including first and last names. Participants were asked to recognize the correct option for the target picture.

#### Recalling of Words Under Conditions of Focused and Divided Attention

Participants completed the test of free recall of word lists under conditions of focused and divided attention tasks. Four lists of 12 words were randomly presented in a predetermined order of conditions: with the simultaneous task of sorting cards during encoding, during retrieval only, during both encoding and retrieval, and during neither encoding nor retrieval. The number of words recalled under each condition was used in data analyses.

### Semantic Memory

#### Word Fluency

Participants were asked to name as many words as possible in specific criteria in 1 min for this test. Five criteria for naming words are presented below. The number of words named correctly in each test was the index of word fluency.

*1. words starting with the letter “a”*

*2. five-letter words starting with the letter “t”*

*3. four-letter words starting with the letter “b”*

*4. professions starting with the letter “m”*

*5. four-letter bird names*

### Statistical Analyses

Memory scores were first transformed to standard z-scores and then averaged for each subscale. We used Pearson correlation, partial correlations, and moderation analysis to test hypotheses. The moderation analysis was conducted using the Hayes approach and PROCESS Macro by performing a bias-corrected bootstrap procedure of 5,000 replications (67).

## Results

### Correlation Analysis

The correlation and partial correlation between the LEQ subscales and memory performance are shown in Table 3. The LEQ total score was correlated with semantic memory (*r* = 0.304, *p* < 0.01) and episodic memory (*r* = 0.439, *p* < 0.01); Knowledge have a significant positive relationship with both semantic (*r* = 0.480, *p* < 0.01) and episodic memory (*r* = 0.412, *p* < 0.01). Also, Physical, Socialization, Artistic, and Leisure subscales were only related to episodic memory (r's 0.178−0.255). Regarding life stages, specific and non-specific activities of the three stages had a significant positive correlation with episodic memory (*r*s 0.189−0.518). Also, all stages, except for specific mid-life activities, had a significant relationship with semantic memory (*r*s 0.240–0.364).

**Table 3:** Correlations and partial correlations with adjustment for age, educational level, and gender between the LEQ and memory performance (*n* = 149).

			**Correlations**		**Partial correlations**	
		**LEQ subscales**	**Semantic memory**	**Episodic memory**	**Semantic memory**	**Episodic memory**
LEQ	5-factor structure	Knowledge	0.480^**^	0.412^**^	0.413^**^	0.369^**^
		Physical	0.064	0.208^*^	0.000	0.114
		Socialization	0.133	0.255^**^	0.052	0.184^*^
		Artistic	0.124	0.178^*^	0.092	0.145
		Leisure	0.100	0.180^*^	−0.010	0.009
		LEQ total score	0.304^**^	0.439^**^	0.173^*^	0.294^**^
	Original structure	YA specific	0.287^**^	0.265^**^	0.118	0.139
		YA non-specific	0.276^**^	0.317^**^	0.198^*^	0.215^**^
		ML specific	0.113	0.189^*^	0.071	0.144
		ML non-specific	0.355^**^	0.518^**^	0.266^**^	0.417^**^
		LL specific	0.364^**^	0.344^**^	0.174^*^	0.269^**^
		LL non-specific	0.240^**^	0.248^**^	0.161	0.139

** *p < 0.01*;

* *p < 0.05*.

*LEQ, The Lifetime of Experiences Questionnaire*.

When adjusted for age, educational level, and gender, Knowledge subscale was correlated with semantic (*r* = 0.413, *p* < 0.01) and episodic memory (*r* = 0.369, *p* < 0.01). In addition, the Socialization subscale was significantly correlated with the episodic memory (*r* = 0.184, *p* < 0.05). Other types of activities did not show a significant relationship with memory. Regarding life stages, YA non-specific, ML non-specific, and LL specific had significant relationships with memory even after controlling age, educational level, and gender (*r's* 0.174–0.417).

### Moderation Analysis

**Figure 2:**
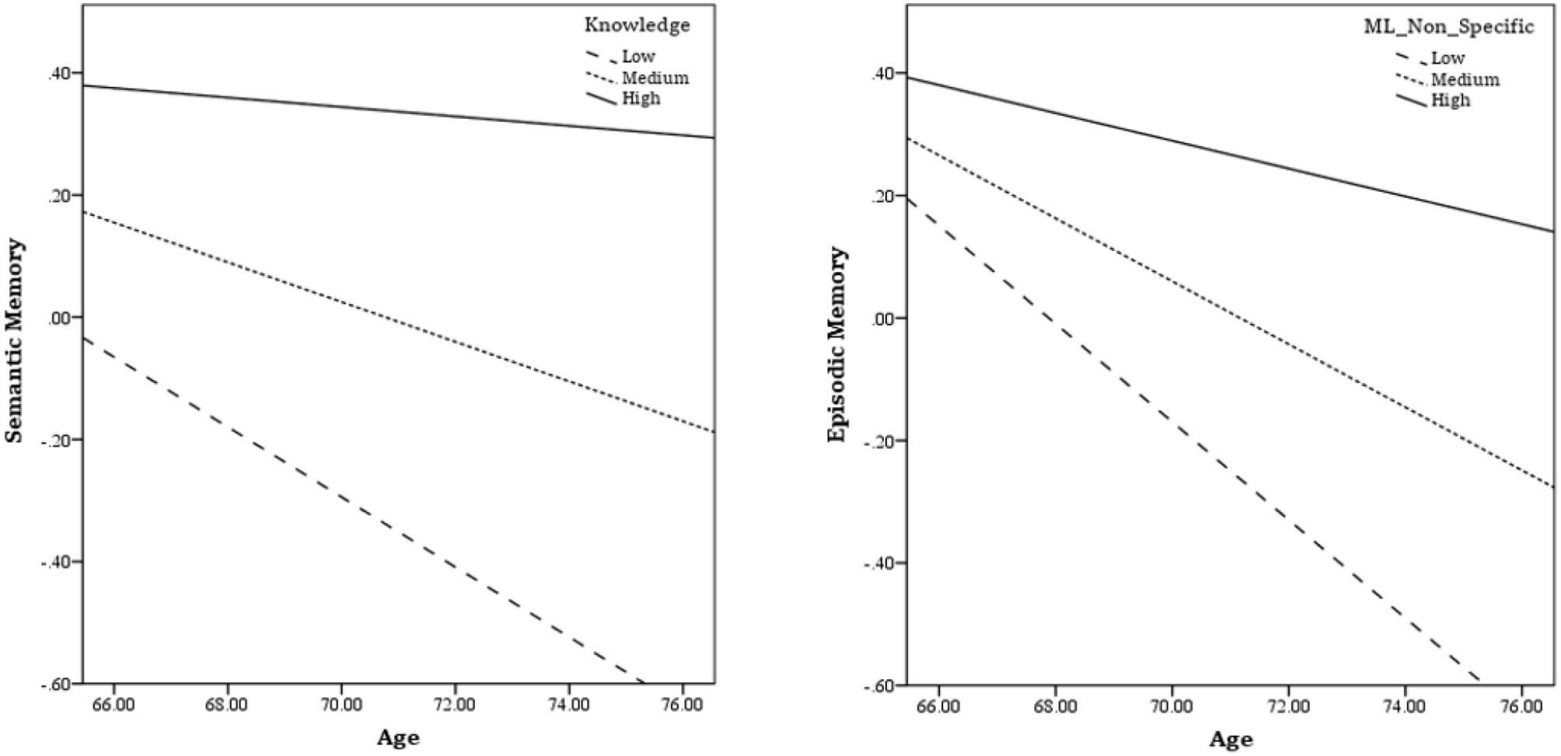
Simple slopes plots for the effect of age on memory at high, medium, and low levels of activities.

This analysis investigated the moderating effect of various activities and experiences of life stages in the relationship between age and memory. The results showed that the moderating effect of the Knowledge component on semantic memory (B = 0.005, SE = 0.002, *t* = 2.021, *p* = 0.045) and the moderating effect of Non-specific mid-life activities on episodic memory (B = 0.007, SE = 0.002, *t* = 3.348, *p* = 0.001) are significant. Other moderating effects were not significant. This interactive effect becomes more apparent when checking out the effect of age on memory at different levels of a moderator variable. Simple slopes analysis indicated that age negatively predicts semantic memory at the low level of Knowledge (B = −0.057, SE = 0.016, *t* = −3.468, *p* = 0.001) and the medium level of Knowledge (B = −0.032, SE = 0.011, *t* = −2.962, *p* = 0.004), but does not predicts at the high level of Knowledge (B = −0.008, SE = 0.016, *t* = −0.468, *p* = 0.640) (Figure 2). This result suggests that the rate of decline in semantic memory becomes slower in participants who have more knowledge activity. The effect of age on semantic memory loses its significance at the high level of knowledge activity.

The interactive effect of non-specific mid-life activities on episodic memory was also significant. The simple slope regarding this analysis suggested that the rate of episodic memory decline becomes slower as non-specific mid-life activities increase. The results showed that age can inversely predict episodic memory performance at the low level (B = −0.080, SE = 0.011, *t* = −7.311, *p* = 0.001), and the medium level of non-specific mid-life activities (B = −0.051, SE = 0.009, *t* = −5.608, *p* = 0.001), but it does not predict at the high level of non-specific mid-life activities (B = −0.023, SE = 0.014, *t* = −1.625, *p* = 0.106) (Figure 2).

## Discussion

The present study first evaluated the factor structure and psychometric properties of the Persian version of the Lifetime of Experiences Questionnaire (LEQ) in the normal elderly. Next, we examined the relationship between lifespan activities and memory in late-life using the LEQ. In general, our results suggested that the Persian version of LEQ is a valid tool for assessing various activities and experiences throughout life. The study also confirmed a positive correlation between the LEQ subscale and memory test scores, suggesting that activities in life can influence memory functioning in late life.

LEQ organizes different experiences and activities of individuals based on life periods. To our knowledge, only one study addressed the categorization of LEQ based on content aspects in the literature (26). In Gonzales' study using factor analysis, nine factors were extracted. These factors included Artistic, Information about the surroundings, Bilingualism, Reading, Musical instruments, Physical activities, social engagement, Social integration and Network, and Economic. The factors extracted in the present study are somewhat similar to those of Gonzales' analysis, with the difference that the number of factors has decreased. Our result suggests that the LEQ has five subscales reflecting the major categories of activities.

The first factor, Knowledge, assessed the frequency of reading activity among older adults, use of a second language, and education. Such activities were considered cognitive activities that lower the risk of dementia among older adults (68–70). The second factor, Physical activity, deals with the frequency and regularity of performing mild to vigorous physical activities throughout life. In addition, late-life working was included in this subscale. In some occupations, the duration of physical activity at work will easily replace exercise in leisure time. Thus, individuals who work at an older age may experience the beneficial effects of physical activity. Furthermore, physical activities enhance the cognitive functions of older adults (71–73).

The third factor, Socialization, is related to visiting friends and family, volunteer activities, and participating in social clubs. All items on this scale include activities requiring social situations and communication with others. Studies show that social networks and engagement protect older adults from cognitive decline (74, 75). The fourth factor, named Artistic, includes playing music and artistic pastimes. That measured the frequency of learning or using art as a leisure activity. Artistic pastimes are supposed to significantly influence the cognitive functions of older adults (76, 77). Finally, the fifth factor measures Leisure activities such as travel, entertainment, and activity of daily living in late life. The effects of Leisure activities are similar to the effects of the other five factors, which improve the cognitive functions of older adults (78, 79). Three items in the LEQ did not belong under any factors. These items include Sources of information about the world and national events in old age (LL_ Information Sources), job rank in middle age (ML_ Occupation Rank), and supervisory in mid-life (ML_ Supervisory Role). This finding is consistent with Gonzales (26) study, in which the ML_ Occupation Rank and ML_ Supervisory Role were not included under any factor.

Reliability analyses demonstrate that the LEQ is internally consistent. The results showed that when using Cronbach's alpha (α), the internal consistency of the Artistic and Leisure subscales is unacceptable. As a consequence of the multidimensionality of the LEQ, its internal consistency may have been underestimated for some subscales. However, when using the mean interitem correlation (MIC) that is not affected by the number of items, the LEQ subscales had adequate internal consistency. Our results regarding the internal consistency of the LEQ subscales are consistent with prior work (24, 26).

Regarding the convergent and discriminant validity of the LEQ scores, the results of this study are in line with prior research and show that LEQ is negatively related to depression in aging (35). Among the subscales of depression, Leisure exhibited the most negative association with depression. This subscale mainly includes hobbies, pastimes, and daily activities of old age. It can be assumed that participating in a wide range of leisure activities may protect against depression in older adults. This finding is consistent with previous research that showed that increasing leisure activity levels might help lower depressive symptoms in older adults (80–82).

In the second study, we investigated the relationship between LEQ subscales and memory function in old age. The results indicated that among the content subscales, Knowledge had the most strongest relationship with both semantic and episodic memory. This relationship was also significant after controlling for age, gender, and educational level. Other subscales were correlated significantly only with episodic memory. The subscale of Knowledge mainly includes the reading and use of a second language, which many studies have supported their relationship with various types of memory. Results revealed that the older adults who participate in reading books have reduced chances of developing cognitive deterioration (68, 69, 83) and perform better in verbal Fluency (84–86) and episodic recall tasks (86, 87). Also, learning and using a second language can contribute to more cognitively and mentally healthy years in older adulthood (88–91) and enhance brain neuroplasticity in aging (92, 93). Therefore, reading materials and learning a second language may represent potentially helpful cognitive activities for promoting healthy aging.

In terms of life stages, ML non-specific has the highest correlation with memory. We found that ML non-specific activities make a unique contribution to late-life memory performance after controlling for age, gender, and educational level. Previous studies on the LEQ have also supported the prominent role of mid-life activities in the cognitive function of the elderly (28, 32). Similarly, Gow et al. (94) found that mid-life intellectual and social activities, but not physical activity, were associated with late-life cognitive health. The relationship of other life stages was significant; Except for the correlation of ML specific with semantic memory. To summarize, lifetime experiences, particularly mid-life activities, can play a pivotal role in the cognitive health of the elderly.

The results of the moderation analysis indicated essential findings regarding the protective role of life activities against memory loss. We examined the moderating effect of life stages and content subscales on the relationship between age and memory. The results showed that the Knowledge and ML non-specific subscales moderate the effect of age on semantic and episodic memory, respectively. The knowledge subscale includes items with the same themes at different stages of life; Conversely, ML non-specific covers various items in mid-life. This finding shows that content aspects are important in the decline of semantic memory; While for episodic memory, structural and temporal details are more important. This view is in line with the difference between semantic and episodic memory that has been proposed in the theoretical background. Episodic memory contains specific information about when and where they were formed, whereas semantic memory lacks such contextual information and is involved in encoding and retrieving general knowledge without a specific time or place reference (95, 96).

Another explanation for the difference in the impact of activities on semantic and episodic memory can be the distinction in their trajectories of cognitive decline. Longitudinal studies show a relatively stable performance level up to middle age, followed by a sharp decline in episodic memory (97). In other words, episodic memory decline begins after middle age, and it can be expected that pre-decline activities in middle age can preserve cognitive function in old age. Higher levels of pre-decline activities protect against age-related pathological changes and take longer to reach any impairment threshold. The difference in the active vs. inactive individual's episodic memory performance on old age might be considered the cognitive reserve associated with the midlife activity.

On the other hand, semantic memory represents an individual's cumulative knowledge about the world (98) and provides dynamic storage of the learning and experience throughout life. Thus, there is a relatively constant performance level across the adult life span for semantic memory (97). This relatively constant pattern of change over time, which lacks sharp decline, minimizes the effect of life stages on semantic memory. In general, episodic memory is more affected by life stages than semantic memory. In contrast, semantic memory is more dependent on the knowledge-based content of activities, regardless of life stages.

Despite these results, the present study is not devoid of limitations. First, we used non-probabilistic sampling for convenience, which may influence the external validity of the research. Second, the cross-sectional design of this study prevented drawing a clear conclusion on the causal relationships between life activities and memory. Future longitudinal studies are needed to confirm their causality. Finally, the LEQ is primarily used as a self-report questionnaire and relies on recall of prior activities, which increases the possibility that cognitively healthy individuals might recall more activities throughout their lives. Therefore, this may restrict access to an unbiased picture of the individual's lifetime activities.

All these taken into account, our results indicated that the Persian LEQ holds promise as a measure to be used by practitioners, healthcare professionals, and policymakers in the aging context. The LEQ may perform as a screening instrument for health professionals to evaluate the different mental activities of older adults. As a screening instrument, professionals can use it as a guide in individually planning their interventions to keep or enhance mental activities. Furthermore, the LEQ may be a promising tool in evaluating cognitive decline and could be helpful in clinical settings to identify people at risk for developing cognitive impairment. The second study's findings also showed that life experiences as a cognitive reserve differentially affect cognitive domains. In particular, the process and content of these experiences affect different aspects of memory. Therefore, the content and variety of activities and life period are three critical components that need to be considered in lifestyle interventions to reduce the risk of dementia.

## Conclusion

To conclude, the Persian version of the LEQ has good psychometric properties and is a valid instrument. Current findings suggest that this tool is a comprehensive instrument for assessing the life activities that may affect cognitive performance in older adults. The LEQ can be a beneficial instrument both for psychological research and for use in clinical and health settings in the context of aging. In addition, Our study demonstrates that Knowledge-based activities throughout life play a protective role in preventing semantic memory impairment in late life. On the other hand, A broad and various range of activities during mid-life can protect the episodic memory performance of the elderly against the destructive effects of aging. These activities reflect lifestyle preferences and are therefore amenable to modification and may have implications for the primary prevention of dementia.

## Data Availability Statement

The raw data supporting the conclusions of this article will be made available by the authors, without undue reservation.

## Ethics Statement

Ethical review and approval was not required for the study on human participants in accordance with the local legislation and institutional requirements. All participants provided their written informed consent to participate in this study.

## Author Contributions

HK gathered data, performed the statistical analysis, and prepared the manuscript. HK and JH designed the study. JH, RR, and AM reviewed and revised the manuscript. All authors interpreted the data and approved the final version of the manuscript.

## Conflict of Interest

The authors declare that the research was conducted in the absence of any commercial or financial relationships that could be construed as a potential conflict of interest.

## Publisher's Note

All claims expressed in this article are solely those of the authors and do not necessarily represent those of their affiliated organizations, or those of the publisher, the editors and the reviewers. Any product that may be evaluated in this article, or claim that may be made by its manufacturer, is not guaranteed or endorsed by the publisher.
